# The American Academy of Dental Surgery

**Published:** 1875-09

**Authors:** 


*The American Academy of Dental Surgery of New York.*—A new society under the above name has been instituted in New York, similar to the Boston "Academy of Dental Science," in its object, and is officered as follows: President.—Geo. H, Perine; Vice-President.—Alfred P. Merrill; Cor. Secretary.—Frederick S. Howard; Rec. Secretary.—N. M. Beckwith; Treasurer.—R. P. Perry; Curator.—John P. Adams; Librarian.—George S. Meigs.

If the members of this new society are equal to their officers in reputation and character, as we have no doubt they are, by taking an active interest in its welfare, such an association must be productive of great results. Their object as set forth in the Charter, is to extend the knowledge of Dental Surgery, to cultivate and develop closer professional relations among its members at home and abroad, to draw out and mature the latest talent evinced by the young and rising members of the profession, and to secure a higher appreciation of the profession, and advance the standard of Dental Science to that position of honor and dignity to which it is so surely entitled.

---

